# The prognostic and predictive effect of body mass index in hormone receptor-positive breast cancer

**DOI:** 10.1093/jncics/pkad092

**Published:** 2023-11-22

**Authors:** Senna W M Lammers, Sandra M E Geurts, Irene E G van Hellemond, Astrid C P Swinkels, Carolien H Smorenburg, Maurice J C van der Sangen, Judith R Kroep, Hiltje de Graaf, Aafke H Honkoop, Frans L G Erdkamp, Wilfred K de Roos, Sabine C Linn, Alexander L T Imholz, Marjolein L Smidt, Ingeborg J H Vriens, Vivianne C G Tjan-Heijnen

**Affiliations:** Department of Medical Oncology, Maastricht University Medical Centre, GROW, Maastricht University, Maastricht, the Netherlands; Department of Medical Oncology, Maastricht University Medical Centre, GROW, Maastricht University, Maastricht, the Netherlands; Department of Medical Oncology, Catharina Hospital, Eindhoven, the Netherlands; Clinical research department, Netherlands Comprehensive Cancer Organisation (IKNL), Nijmegen, the Netherlands; Department of Medical Oncology, Netherlands Cancer Institute, Amsterdam, the Netherlands; Department of Radiation Oncology, Catharina Hospital, Eindhoven, the Netherlands; Department of Medical Oncology, Leiden University Medical Centre, Leiden, the Netherlands; Department of Medical Oncology, Medical Centre Leeuwarden, Leeuwarden, the Netherlands; Department of Medical Oncology, Isala Clinics, Zwolle, the Netherlands; Department of Medical Oncology, Zuyderland Medical Centre Heerlen-Sittard-Geleen, location Sittard-Geleen, the Netherlands; Department of Surgery, Gelderse Vallei Hospital, Ede, the Netherlands; Department of Medical Oncology, Netherlands Cancer Institute, Amsterdam, the Netherlands; Department of Pathology, University Medical Centre Utrecht, Utrecht, the Netherlands; Department of Medical Oncology, Deventer Hospital, Deventer, the Netherlands; Department of Surgery, Maastricht University Medical Centre, GROW, Maastricht University, Maastricht, the Netherlands; Department of Medical Oncology, Maastricht University Medical Centre, GROW, Maastricht University, Maastricht, the Netherlands; Department of Medical Oncology, Maastricht University Medical Centre, GROW, Maastricht University, Maastricht, the Netherlands

## Abstract

**Background:**

Obesity has been associated with an adverse prognosis and reduced efficacy of endocrine therapy in patients with hormone receptor-positive (HR+) breast cancer (BC). This study determines the prognostic and predictive effect of body mass index (BMI) on the disease-free survival (DFS) of postmenopausal HR+ BC patients.

**Methods:**

Patients were identified from the DATA study (NCT00301457), a randomized controlled trial evaluating the efficacy of 6 vs 3 years of anastrozole after 2 to 3 years of adjuvant tamoxifen in postmenopausal women with HR+ BC. Patients were classified as normal weight (BMI: 18.5–24.9 kg/m^2^), overweight (25.0–29.9 kg/m^2^), or obese (≥30.0 kg/m^2^). The primary endpoint was DFS, evaluated from randomization (prognostic analyses) or 3 years after randomization onwards (predictive analyses; aDFS) using multivariable Cox regression analyses. *P*-values were 2-sided.

**Results:**

This study included 678 normal weight, 712 overweight, and 391 obese patients. After a median follow-up of 13.1 years, overweight and obesity were identified as negative prognostic factors for DFS (hazard ratio (HR) = 1.16; 95% confidence interval (CI) = 0.97 to 1.38 and HR = 1.26; 95% CI = 1.03 to 1.54, respectively). The adverse prognostic effect of BMI was observed in women aged younger than 60 years, but not in women aged 60 years or older (*P*-interaction = .009). The effect of extended anastrozole on aDFS was similar in normal weight (HR = 1.00; 95% CI = 0.74 to 1.35), overweight (HR = 0.74; 95% CI = 0.56 to 0.98), and obese patients (HR = 0.97; 95% CI = 0.69 to 1.36) (*P*-interaction = .24).

**Conclusion:**

In this study among 1781 HR+ BC patients, overweight and obesity were adverse prognostic factors for DFS. BMI did not impact the efficacy of extended anastrozole.

One in five women worldwide are estimated to be obese by 2025 (1). Obese patients are more likely to develop comorbidities, such as diabetes mellitus, cardiovascular disease, and several types of cancer (2). Obesity has also been associated with an increased risk of hormone receptor-positive (HR+) breast cancer (BC) in postmenopausal women and an adverse prognosis after BC diagnosis (3-8). Potential mechanisms for this elevated risk and adverse prognosis include an increased peripheral conversion of androgens to estrogens in adipose tissue, higher leptin concentrations, hyperinsulinemia, and obesity-mediated inflammation (6).

In the general population, however, the association between obesity and all-cause mortality tends to differ between younger and older adults (9). The majority of studies observed no adverse association between obesity and all-cause mortality in adults aged 65 years or older (10-13). In patients with BC, the association between obesity and outcomes may also differ by age or menopausal status (7,14,15). In a large meta-analysis of 82 studies including 213 075 BC survivors, for example, numerically stronger associations between obesity and all-cause mortality and breast-cancer-specific mortality (BCSM) were observed in premenopausal versus postmenopausal women (7). In addition, two studies observed an inverse association between a higher body mass index (BMI) and all-cause mortality in older BC patients, although results were inconclusive for BCSM (14,15).

Over the years, the impact of BMI on the efficacy of (extended) endocrine therapy has also been studied in postmenopausal women with HR+ BC. Tamoxifen seems to be equally effective across BMI classes (16). However, conflicting results have been reported on the association between BMI and the efficacy of aromatase inhibitors (17-19). The ATAC trial, for example, observed a trend towards a reduced benefit of 5 years of anastrozole versus 5 years of tamoxifen in patients with a higher BMI (17). In addition, the ABCSG-6a trial observed a benefit of 3 additional years of anastrozole after 5 years of tamoxifen in normal weight patients only, while no benefit was observed in overweight or obese patients (19). However, in the BIG 1-98 trial, BMI did not affect the efficacy of 5 years of letrozole versus tamoxifen (18).

The present post hoc study was performed within the framework of the DATA study, a randomized controlled trial that evaluated the efficacy of 6 versus 3 years of anastrozole after 2 to 3 years of adjuvant tamoxifen in postmenopausal women with HR+ BC (20,21). The primary aim of this exploratory analysis was to explore the association between BMI and disease outcomes in the DATA study cohort as a whole and by age subgroups. The secondary aim was to explore the association between BMI and the efficacy of extended anastrozole therapy.

## Methods

### Study design and participants

The DATA study (NCT00301457) was an open-label, phase 3, randomized controlled trial in which postmenopausal women with HR+ BC received either 6 or 3 years of anastrozole (1 mg orally once a day) after completing 2 to 3 years of adjuvant treatment with tamoxifen without signs of disease recurrence (20). From 2006 to 2009, 1912 patients were recruited from 79 hospitals in the Netherlands and screened for eligibility. The final study population consisted of 1860 patients, of whom 1660 were disease-free at 3 years after randomization. The main efficacy results have been published elsewhere (20, 21).

For the current analysis, all patients with a baseline BMI measurement were selected. Underweight patients (BMI: <18.5 kg/m^2^) were excluded because of the small number of patients.

This study was approved by the medical ethics committee of the Radboud University Medical Centre (Nijmegen, the Netherlands). Written informed consent was obtained from all patients.

### Data collection and definitions

Height and weight were measured by the treating physician or self-reported by the patient at randomization and were used to calculate BMI. We categorized BMI according to the World Health Organization criteria: normal weight (BMI: 18.5-24.9 kg/m^2^), overweight (BMI: 25.0-29.9 kg/m^2^), or obese (BMI: ≥30.0 kg/m^2^). Follow-up was performed by the treating physician every 6 months during the first 6 years after randomization and yearly thereafter. A mammogram was done once a year. Database lock: March 7, 2022.

### Endpoints

The primary endpoint was disease-free survival (DFS). Secondary endpoints were overall survival (OS), BCSM, and other-cause mortality (OCM). The following events ended a period of DFS: BC recurrence, second primary BC, second primary cancer (excluding basal cell or squamous cell carcinoma of the skin and carcinoma in situ of the cervix), or death from any cause. A period of OS ended as a result of death from any cause. All BC-related deaths were included in the analysis of BCSM, whereas all non-BC-related deaths were included in the analysis of OCM.

### Statistical analysis

Baseline characteristics were compared by BMI class and assigned treatment. The chi-squared test and the Mann-Whitney U test were used to assess differences in categorical or continuous variables. The presence of a trend was evaluated using the Cochran-Mantel-Haenszel test and the Jonckheere-Terpstra test.

The prognostic effect of BMI was evaluated irrespective of assigned treatment from date of randomization onwards. The predictive effect of BMI on the efficacy of 6 versus 3 years of anastrozole was assessed from 3 years after randomization onwards (ie, ‘adapted’ endpoints). Patients with a DFS event or patients who were lost to follow-up within 3 years after randomization were excluded from the adapted analyses.

DFS and OS were examined using Kaplan-Meier survival analyses and Cox regression analyses, when adjusting for potential confounders. BCSM and OCM were determined with competing risk methodology, using the Fine and Gray model when adjusting for potential confounders. Differences between BMI classes and treatment groups were assessed with the log-rank test and the Gray’s test. In the absence of an event, patients were censored at the last follow-up visit in all analyses. Death not related to BC was considered a competing event in the analysis of BCSM. BC-related death was considered a competing event in the analysis of OCM. Missing data of confounders were imputed.

Prognostic analyses were stratified by age (<60 versus ≥60 years). The cutoff point of 60 years, ie, 57 years at BC diagnosis, was chosen to distinguish younger postmenopausal women from older postmenopausal women, as some younger patients were premenopausal at BC diagnosis (22,23). The BMI-by-age and treatment-by-BMI interaction terms were calculated using likelihood-ratio tests.

*P*-values were 2-sided and considered statistically significant at a value of .05 or less.

All statistical analyses were performed with SPSS (version 25) and Stata (version 17).

## Results

### Patient characteristics

**Figure 1.**
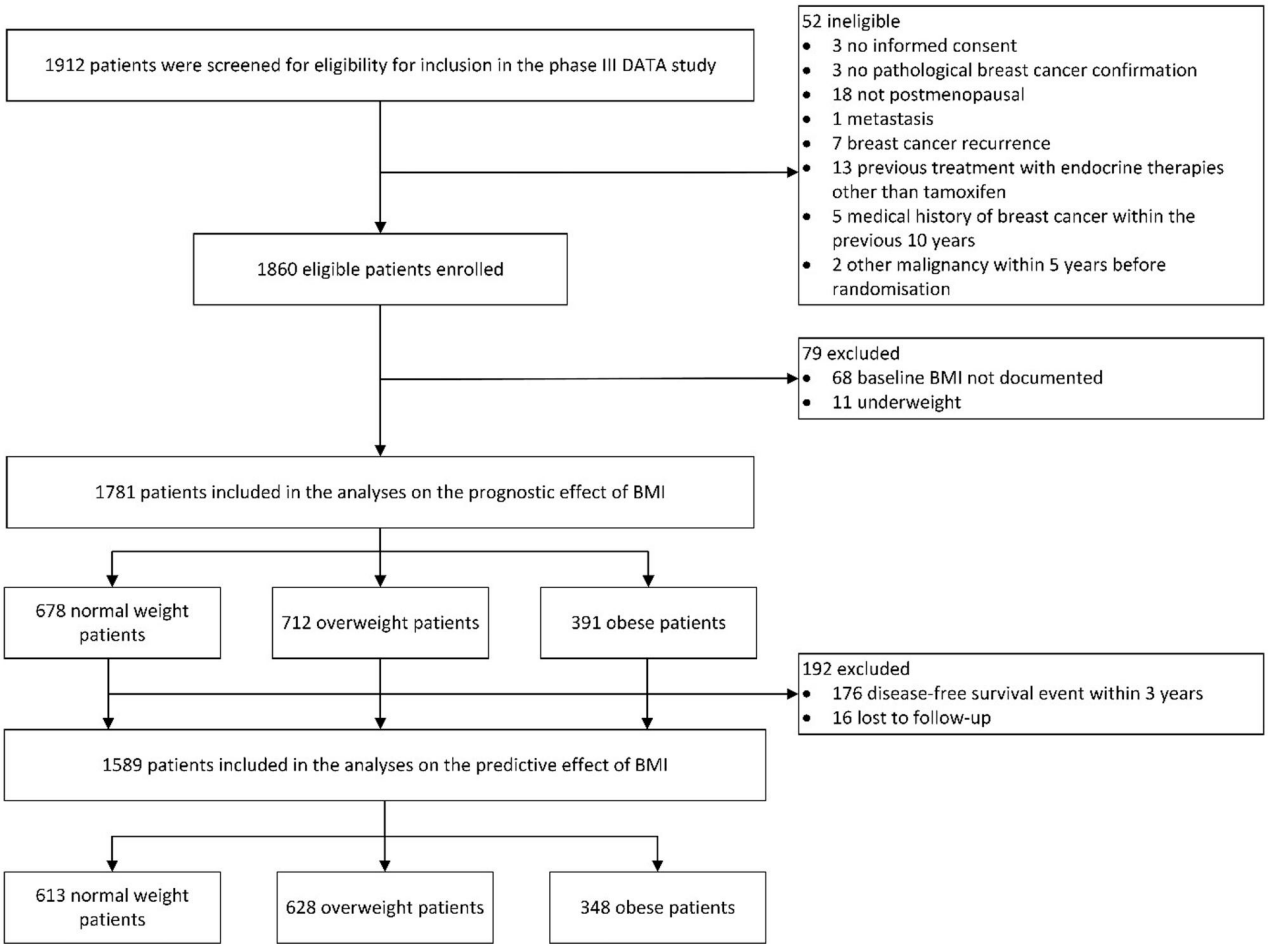
Flowchart of included patients. BMI = body mass index.

Overall, 1781 patients were included in the analysis on the prognostic effect of BMI (Figure 1). Of these, 678 (38.0%) were normal weight, 712 (40.0%) overweight, and 391 (22.0%) obese at randomization. A higher BMI class was associated with higher age, presence of cardiovascular disease, and higher tumor stage (Table 1). In the total study population, the use of (neo)adjuvant chemotherapy decreased with increasing BMI. However, in the stratified analyses by age, the association between BMI and (neo)adjuvant chemotherapy disappeared (Supplementary Tables 1 and 2, available online).

**Table 1.** Baseline characteristics of study participants according to body mass index class (No. [%])^a^^,^^b^

Characteristic	Normal weight (n = 678)	Overweight (n = 712)	Obese (n = 391)	*P* for trend
Median age at randomization				<.001
Years (IQR)	54 (50–62)	59 (52–65)	61 (53–66)	
History of cardiovascular disease				<.001
Yes	147 (22)	253 (36)	204 (52)	
Smoking history				.89
Nonsmoker	311 (47)	342 (49)	182 (47)	
Previous or current smoker	348 (53)	357 (51)	203 (53)	
Tumor stage				.02
pT1	330 (49)	306 (43)	159 (41)	
pT2	289 (43)	355 (50)	196 (50)	
pT3/4	57 (8)	51 (7)	36 (9)	
Nodal status				.98
Negative	218 (32)	237 (33)	125 (32)	
Positive	460 (68)	475 (67)	266 (68)	
Histologic grade				.56
Grade I	122 (19)	113 (16)	68 (18)	
Grade II	326 (50)	367 (53)	197 (53)	
Grade III	211 (32)	217 (31)	106 (29)	
Hormone receptor status				.79
ER and PR positive	517 (76)	521 (73)	298 (76)	
ER or PR positive	161 (24)	191 (27)	93 (24)	
HER2 status				.79
Positive	15 (2)	20 (3)	9 (3)	
Negative	618 (98)	635 (97)	349 (98)	
Histology				.71
Lobular	135 (20)	104 (15)	87 (22)	
Other	543 (80)	608 (85)	304 (78)	
Breast-conserving surgery				.63
Yes	331 (49)	351 (49)	197 (50)	
Prior (neo)adjuvant chemotherapy				.002
Yes	485 (72)	478 (67)	245 (63)	
Median previous duration of tamoxifen				.38
Years (IQR)	2.3 (2.1–2.5)	2.3 (2.1–2.5)	2.3 (2.1–2.5)	
Recommended treatment duration of anastrozole				.70
3 years	337 (50)	344 (48)	201 (51)	
6 years	341 (50)	368 (52)	190 (49)	

a Percentages may exceed 100% because of rounding. ER = estrogen receptor; HER2 = human epidermal growth factor receptor 2; IQR = interquartile range; PR = progesterone receptor.

b Missing values: smoking history (n = 38), tumor status (n = 2), histologic grade (n = 54), and HER2 status (n = 135).

### BMI as a prognostic factor

After a median follow-up period of 13.1 years (interquartile range [IQR]: 12.5-13.9) beyond randomization, 706 patients had developed a DFS event and 484 patients had died. Details about endpoint events per BMI class are presented in Table 2.

**Table 2.** Endpoint events in the total study population according to body mass index class (No. [%])

	Normal weight (n = 678)	Overweight (n = 712)	Obese (n = 391)
Event	Number of events (%)		
**Disease-free survival event** ^a^	231	287	188
Recurrence of the primary tumor	112 (48)	133 (46)	97 (52)
Local recurrence	20 (9)	16 (6)	13 (7)
Regional recurrence	25 (11)	24 (8)	16 (9)
Distant recurrence^b^	83 (36)	114 (40)	83 (44)
Visceral	44 (19)	70 (24)	43 (23)
Bone	53 (23)	66 (23)	56 (30)
Soft tissue	10 (4)	21 (7)	15 (8)
Other	3 (1)	1 (<1)	5 (3)
Second, (non-)invasive breast cancer	20 (9)	30 (10)	21 (11)
Second, non-breast cancer	73 (32)	72 (25)	40 (21)
Death without prior breast cancer event	29 (13)	53 (18)	36 (19)
**Death from any cause**	153	204	127
Breast cancer related	79 (52)	103 (51)	65 (51)
Not breast cancer related	62 (41)	74 (36)	49 (39)
Second primary malignancy	35 (23)	40 (20)	18 (14)
Cardiovascular disease	9 (6)	13 (6)	15 (12)
Other	18 (12)	21 (10)	16 (13)
Unknown	12 (8)	27 (13)	13 (10)

a Patients may have had multiple disease-free survival events at the same moment.

b In some patients multiple locations of recurrence were reported.

**Figure 2.**
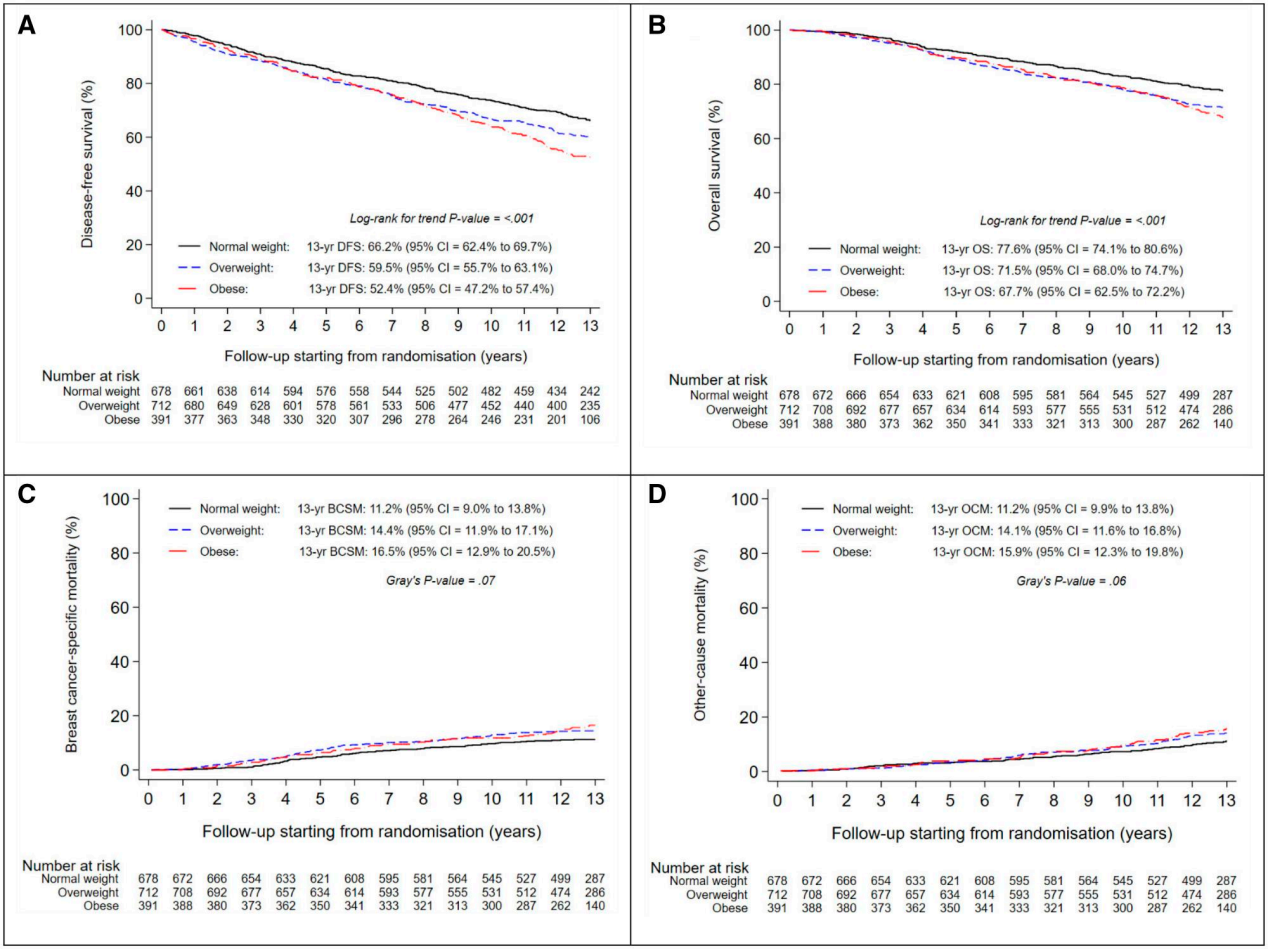
Disease-free survival (A), overall survival (B), breast-cancer-specific mortality (C), and other-cause mortality (D), according to body mass index class at randomization. BCSM = breast cancer-specific mortality; DFS = disease-free survival; OCM = other-cause mortality; OS = overall survival.

The 13-year DFS rates were 66.2% (95% confidence interval [CI] = 62.4% to 69.7%) in normal weight, 59.5% (95% CI = 55.7% to 63.1%) in overweight, and 52.4% (95% CI = 47.2% to 57.4%) in obese patients (Figure 2, A). Overweight and obese patients experienced a deterioration in DFS when compared with normal weight patients (adjusted hazard ratio [HR] = 1.16; 95% CI = 0.97 to 1.38; *P* = .10 and adjusted HR = 1.26; 95% CI = 1.03 to 1.54; *P* = .03, respectively) (Table 3, Supplementary Table 6, available online).

**Table 3.** Multivariable analyses of primary and secondary outcomes according to body mass index class at randomization, overall and stratified by age at randomization

	BMI					
	Normal weight	Overweight		Obese		
Endpoint	Reference	(s)HR^a^ (95% CI)	*P*-value	(s)HR^a^ (95% CI)	*P*-value	*P* interaction
**Disease-free survival**						
All patients (n = 1781 patients, 706 events)	1.00	1.16 (0.97 to 1.38)	.10	1.26 (1.03 to 1.54)	.03	
						.009
<60 years (n = 1023 patients, 323 events)	1.00	1.29 (1.00 to 1.67)	.05	1.83 (1.36 to 2.46)	<.001	
≥60 years (n = 758 patients, 383 events)	1.00	1.04 (0.82 to 1.33)	.72	0.94 (0.72 to 1.23)	.63	
**Overall survival**						
All patients (n = 1781 patients, 484 events)	1.00	1.20 (0.97 to 1.48)	.10	1.16 (0.91 to 1.48)	.23	
						.07
<60 years (n = 1023 patients, 191 events)	1.00	1.46 (1.05 to 2.04)	.03	1.62 (1.09 to 2.42)	.02	
≥60 years (n = 758 patients, 293 events)	1.00	1.02 (0.77 to 1.34)	.90	0.93 (0.68 to 1.26)	.62	
**Breast cancer-specific mortality^b^**						
All patients (n = 1781 patients, 247 events)	1.00	1.25 (0.93 to 1.68)	.15	1.36 (0.97 to 1.91)	.07	
						.56
<60 years (n = 1023 patients, 119 events)	1.00	1.44 (0.96 to 2.18)	.08	1.32 (0.78 to 2.25)	.31	
≥60 years (n = 758 patients, 128 events)	1.00	1.04 (0.67 to 1.60)	.88	1.28 (0.83 to 1.99)	.27	
**Other-cause mortality^b^**						
All patients (n = 1781 patients, 237 events)	1.00	1.12 (0.83 to 1.52)	.45	1.01 (0.71 to 1.43)	.96	
						.02
<60 years (n = 1023 patients, 72 events)	1.00	1.39 (0.79 to 2.45)	.25	2.01 (1.11 to 3.33)	.02	
≥60 years (n = 758 patients, 165 events)	1.00	1.00 (0.70 to 1.44)	.99	0.75 (0.50 to 1.13)	.17	

a The full multivariable models of the total study population are displayed in Supplementary Tables 6 to 9. Analyses were adjusted for age (≥60 years vs <60 years), history of cardiovascular disease (yes vs no), smoking history (yes vs no), tumor status (≥pT2 vs pT1), nodal status (pN positive vs pN negative), histology (lobular vs other), histologic grade (histologic grade 3 vs histologic grade 1 and 2), hormone receptor status (estrogen receptor-positive or progesterone receptor-positive vs estrogen receptor-positive and progesterone receptor-positive), and previous chemotherapy (yes vs no). Age was excluded as a confounding factor in the stratified analyses by age. BMI = body mass index; CI = confidence interval; (s)HR = (subdistribution) hazard ratio.

b In the analyses of breast cancer-specific mortality and other-cause mortality, we reported sHR instead of HR.

The 13-year OS rates were 77.6% (95% CI = 74.1% to 80.6%) in normal weight, 71.5% (95% CI = 68.0% to 74.7%) in overweight, and 67.7% (95% CI = 62.5% to 72.2%) in obese patients (Figure 2, B). When compared with normal weight patients, this resulted in an adjusted HR of, respectively, 1.20 (95% CI = 0.97 to 1.48; *P* = .10) and 1.16 (95% CI = 0.91 to 1.48; *P* = .23) for overweight and obese patients (Table 3, Supplementary Table 7, available online).

Overweight and obese patients also experienced numerically higher BCSM rates when compared with normal weight patients (Figure 2, C). The 13-year cumulative incidence of BCSM was 11.2% (95% CI = 9.0% to 13.8%) in normal weight, 14.4% (95% CI = 11.9% to 17.1%) in overweight, and 16.5% (95% CI = 12.9% to 20.5%) in obese patients. In multivariable analysis, this resulted in a HR of, respectively, 1.25 (95% CI = 0.93 to 1.68; *P* = .15) and 1.36 (95% CI = 0.97 to 1.91; *P* = .07) for overweight and obese patients (Table 3, Supplementary Table 8, available online).

Furthermore, overweight and obese patients had numerically higher OCM rates when compared with normal weight patients (Figure 2, D). However, in multivariable analysis, the cumulative incidence of OCM was similar in overweight and obese patients (HR = 1.12; 95% CI = 0.83 to 1.52; *P* = .45 and HR = 1.01; 95% CI = 0.71 to 1.43; *P* = .96, respectively) (Table 3, Supplementary Table 9, available online).

Age showed to be a statistically significant effect modifier of the association between BMI and DFS (*P*-interaction = .009) and was nearly statistically significant for the association between BMI and OS (*P*-interaction = .07) (Table 3). Specifically, overweight and obese patients aged younger than 60 years experienced a statistically significant deterioration in both DFS and OS when compared with normal weight patients of the same age, whereas no adverse prognostic effect of overweight and obesity was observed in patients aged 60 years or older. The association between BMI and OCM also differed by age (*P*-interaction = .02), but the association between BMI and BCSM was similar in both age groups (*P*-interaction = .56).

### BMI as a predictive factor for benefit of extended endocrine therapy

Overall, 1589 patients were included in the analysis on the predictive effect of BMI: 613 (38.6%) normal weight, 628 (39.5%) overweight, and 348 (21.9%) obese patients (Figure 1). Supplementary Tables 3–5 (available online) present the baseline characteristics according to assigned treatment for every BMI class separately. Obese patients who received 6 years of anastrozole were more frequently diagnosed with a node-positive tumor when compared with those who received 3 years of anastrozole (72% vs 61%, *P* = .04). All other baseline characteristics were equally distributed between treatment groups.

In the total DATA study cohort (n = 1660), 6 versus 3 years of anastrozole resulted in a HR of 0.86 (95% CI = 0.72 to 1.01; *P* = .073) for adapted DFS (aDFS) and a HR of 0.93 (95% CI = 0.75 to 1.16; *P* = .53) for adapted OS (aOS), respectively (21).

**Figure 3.**
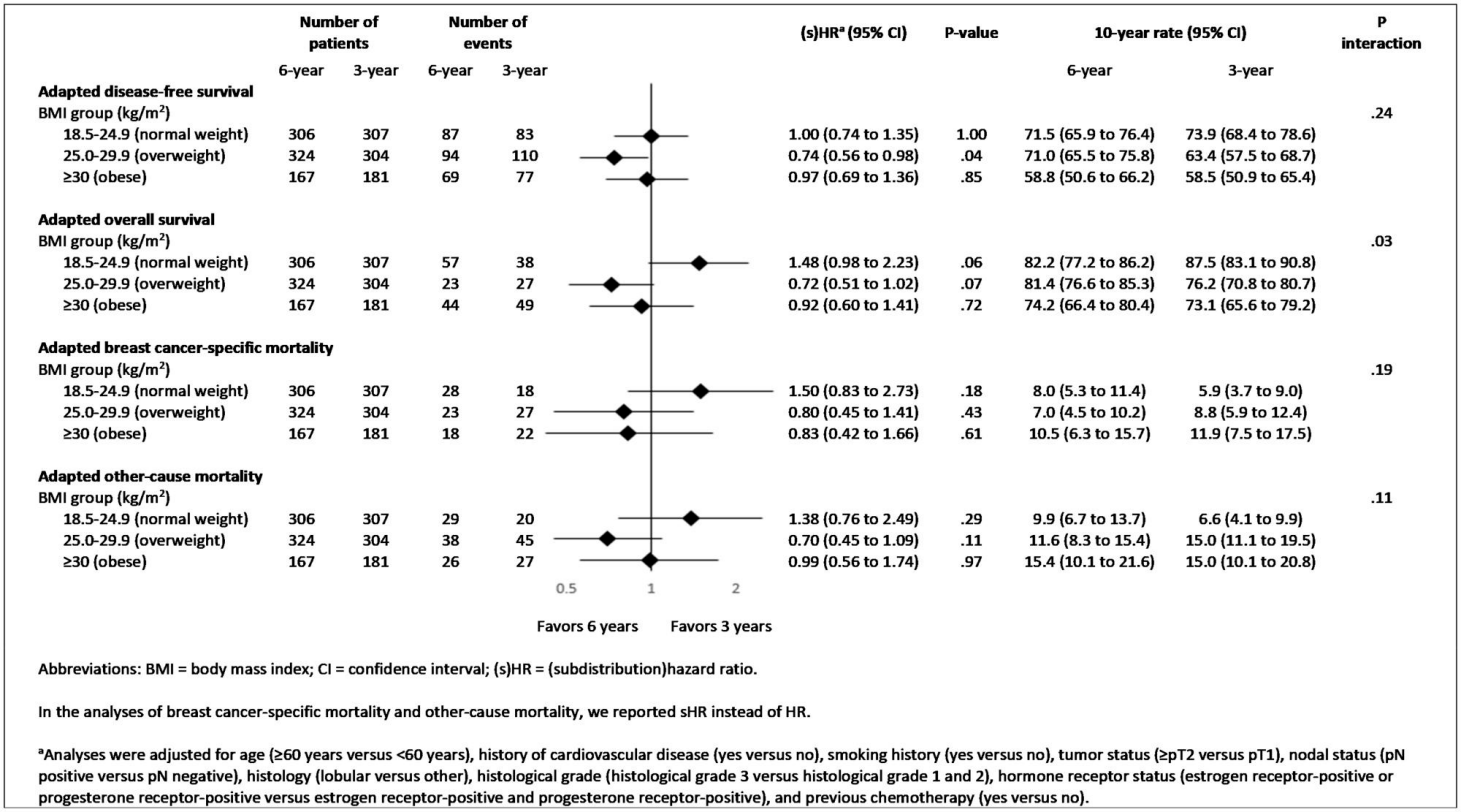
Multivariable analyses of primary and secondary endpoints evaluating the efficacy of 6 vs 3 years of anastrozole, stratified by body mass index class at randomization.

**Figure 4.**
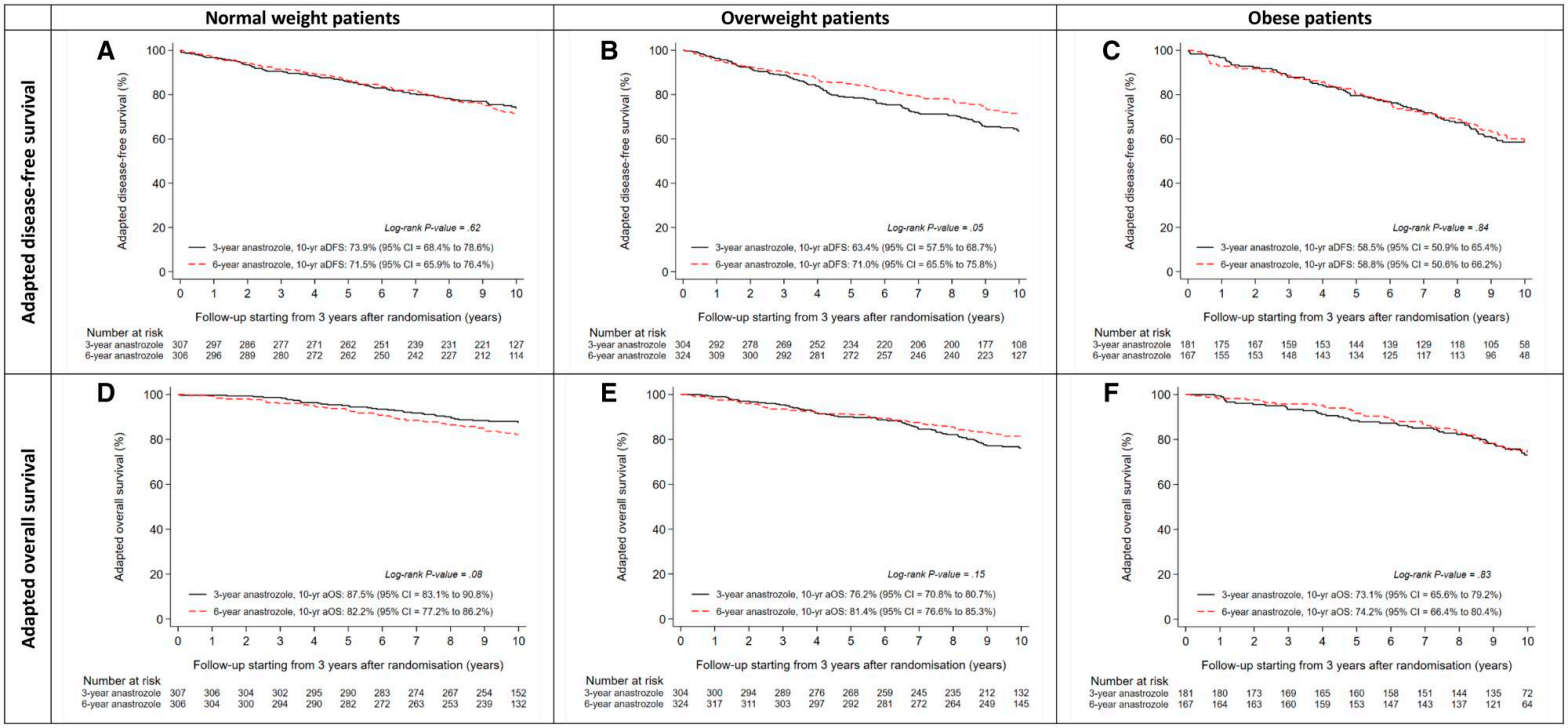
Adapted disease-free survival according to assigned treatment in normal weight (A), overweight (B), and obese (C) patients, and adapted overall survival according to assigned treatment in normal weight (D), overweight (E), and obese (F) patients. aDFS = adapted disease-free survival; aOS = adapted overall survival.

The effect of 6 versus 3 years of anastrozole on aDFS was similar in normal weight (adjusted HR = 1.00; 95% CI = 0.74 to 1.35; *P* = 1.00), overweight (adjusted HR = 0.74; 95% CI = 0.56 to 0.98; *P* = .04), and obese patients (adjusted HR = 0.97; 95% CI = 0.69 to 1.36; *P* = .85) (*P*-interaction = .24) (Figures 3 and 4, A–C).

In the analysis of aOS, the effect of 6 versus 3 years of anastrozole differed between normal weight (adjusted HR = 1.48; 95% CI = 0.98 to 2.23; *P* = .06), overweight (adjusted HR = 0.72; 95% CI = 0.51 to 1.02; *P* = .07), and obese patients (adjusted HR = 0.92; 95% CI = 0.60 to 1.41; *P* = .72) (*P*-interaction = .03) (Figures 3 and 4, D–F). These results did not differ between patients aged younger than 60 years and patients aged 60 years or older (Supplementary Figure 1, available online).

**Figure 5.**
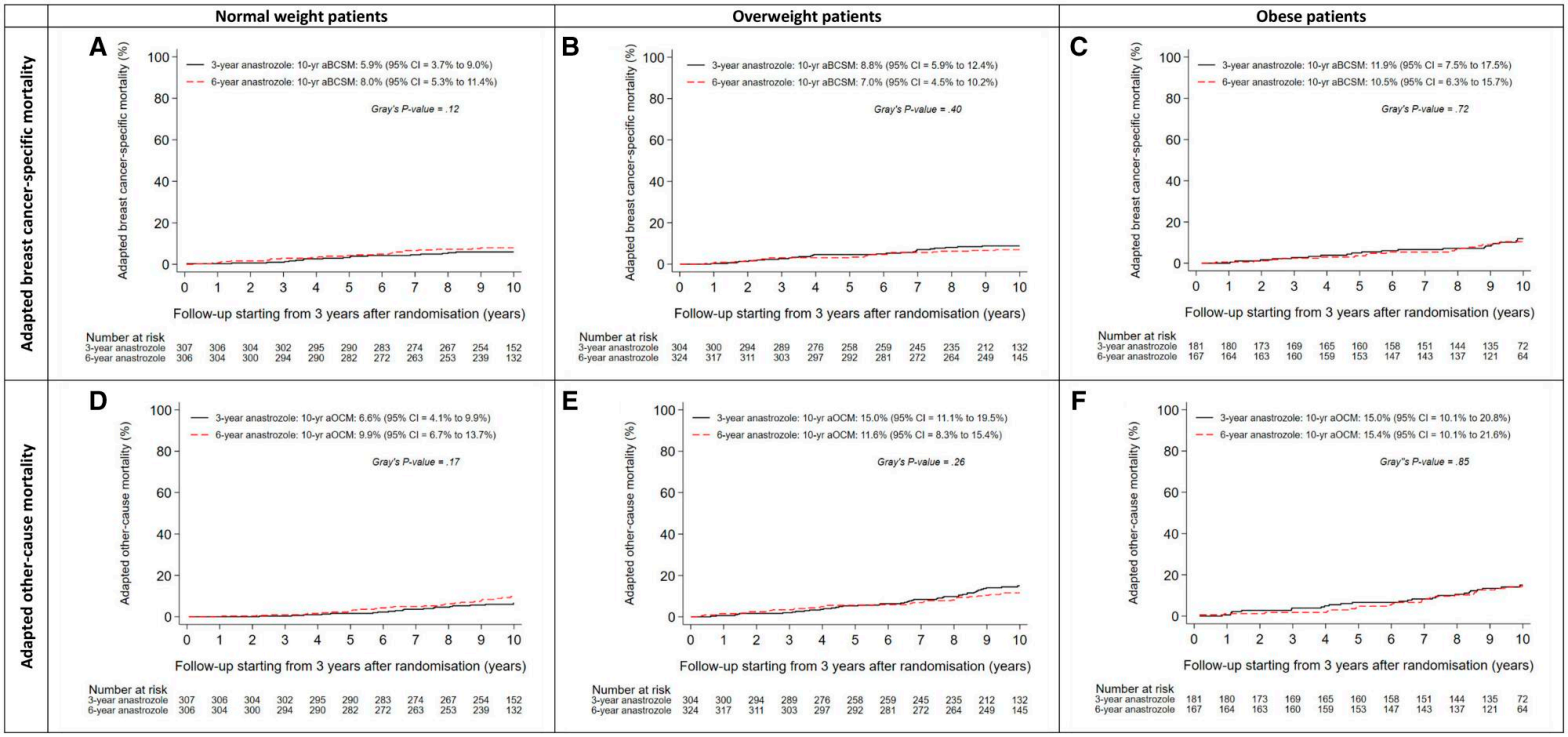
Adapted breast cancer-specific mortality according to assigned treatment in normal weight (A), overweight (B), and obese (C) patients, and adapted other-cause mortality according to assigned treatment in normal weight (D), overweight (E), and obese (F) patients. aBCSM = adapted breast cancer-specific mortality; aOCM = adapted other-cause mortality.

Results of both adapted BCSM (aBCSM) and adapted OCM (aOCM) were comparable to those of aOS in every BMI class (Figures 3 and 5, A–F).

## Discussion

In this study, we evaluated the prognostic and predictive effect of BMI in 1781 postmenopausal women with nonmetastatic HR+ BC from the phase III DATA study. We confirmed the results from previous studies, showing a negative association between obesity and DFS in the overall study population. Interestingly, however, subgroup analyses demonstrated that the negative association between obesity and DFS was statistically significant in patients aged younger than 60 years, but not in patients aged 60 years or older. The effect modification by age is of potential interest.

We observed that obesity was associated with a decrease in DFS and OS in younger postmenopausal HR+ BC patients only. However, while obesity was also associated with an increase in OCM in younger patients only, it seemed to increase the risk of BCSM irrespective of age. These findings indicate that different mechanisms might apply to the association between obesity and OCM and the association between obesity and BCSM. Primarily, the increased risk of OCM in younger obese patients may be the result of developing cardiovascular or metabolic diseases at a younger age (2). In our study, we collected causes of death, but numbers of events per subcategory, ie, cardiovascular death, were too low to perform additional analyses. Secondarily, the lack of an adverse association between obesity and OCM in older patients may be attributed to the “obesity paradox”, which has earlier been described for patients with cancer (24). Potential age-specific explanations for the obesity paradox include a reduced osteoporotic fracture risk due to a higher bone mineral density, reverse causation, and survival bias (10, 12, 25, 26). Reverse causation occurs when previously overweight or obese patients are misclassified as normal weight as a result of disease-related unintentional weight loss right before the BMI measurement. This misclassification may result in an overestimation of the mortality risk in normal weight patients, thereby minimizing the adverse prognostic effect of overweight and obesity in older patients. The obesity paradox does not seem to apply, however, to the association between BMI and BCSM, as we observed that obese patients experience an increased risk of BCSM irrespective of age in our study. This increased risk can be explained by several mechanisms, including higher estrogen levels as a result of increased aromatization in adipose tissue, hyperinsulinemia, and obesity-mediated inflammation (6). The results of our study suggest that these biological mechanisms equally impact the prognosis of younger and older postmenopausal HR+ BC patients with obesity. Nonetheless, the use of BMI has its limitations. BMI does not distinguish between fat and muscle mass and is therefore an inadequate measure of body composition (26). We did not have information about body composition, and in particular the presence of sarcopenia, in our cohort of patients. Several studies have shown, however, that sarcopenia adversely impacts the prognosis of patients with (metastatic) BC (27-30).

In our study, we did not observe a reduced efficacy of extended anastrozole therapy in overweight or obese postmenopausal women with HR+ BC. In the analysis of aOS, however, we did observe a potential difference in treatment effects between BMI classes. In fact, 6 versus 3 years of anastrozole was associated with a non–statistically significantly increased risk of death in normal weight patients (HR = 1.48; 95% CI = 0.98 to 2.23), whereas it was associated with a non–statistically significantly decreased risk of death in overweight patients (HR = 0.72; 95% CI = 0.51 to 1.02). Obviously, as the number of patients and events per subgroup was low, this difference in treatment effects could simply be a chance finding. Alternatively, one might speculate that normal weight patients receiving extended aromatase inhibition are at an increased risk of developing endocrine resistance, as an increased risk of BCSM was also observed. We did not, however, observe a decrease in the DFS of normal weight patients receiving extended aromatase inhibition, although this might also be the result of a decrease in the incidence of second primary cancers in the extended therapy group. In addition, one might speculate that normal weight patients receiving 6 years of anastrozole experience an increased risk of death due to adverse events, ie, cardiovascular events or bone fractures. In a previous report of the DATA study, it was shown that the incidence of cardiovascular events and bone fractures during the first 6 years after randomization did not differ between patients receiving 6 versus 3 years of anastrozole, but the incidence of osteopenia or osteoporosis was higher in the extended therapy group (20). Considering the fact that normal weight patients do not experience an obesity-mediated increase in bone mineral density, it is possible that normal weight patients experience an increased risk of osteoporotic fractures when receiving extended aromatase inhibition. Unfortunately, we do not have data about the incidence of bone fractures after the first 6 years of randomization. Apart from focusing on the increased risk of death in normal weight patients, one might question why overweight patients experienced a reduced risk of death when receiving extended aromatase inhibition in our study. This is an unexpected finding, as both the ATAC trial and the ABCSG-6a trial observed a reduced efficacy of (extended) anastrozole therapy in postmenopausal HR+ BC patients with a higher BMI (17, 19). Furthermore, it is well described that estrogen levels of postmenopausal women increase with a higher BMI, thereby increasing the risk of BC events (6). Therefore, the results of our study should be interpreted with caution until further research on this topic is available.

The major strength of our study is the use of data from patients who participated in a randomized controlled trial, in which endpoints were well defined and consistently measured during follow-up. Another strength of our study is the long-term follow-up period of more than 13 years after randomization. Our study also has some limitations. Patients may experience changes in body weight after BC diagnosis (31-34). We obtained information about BMI at randomization, ie, 2 to 3 years after diagnosis, and we did not collect information about BMI at BC diagnosis. The impact on the study results is, however, expected to be small as a recent meta-analysis by Chan et al. showed that the adverse prognostic effect of obesity on OS remained present, regardless of the moment of BMI measurement (7). Our study also lacked information about diet, physical activity, socioeconomic status, and other factors that may be associated with BMI. The use of self-reported measurements of height and weight in some patients can be considered another limitation of this study. However, in the meta-analysis by Chan et al., the association between obesity and all-cause mortality was similar in studies that used measured versus self-reported values (7). Furthermore, BMI was not a stratification factor in the DATA study (20). The efficacy results of the subgroup analyses by BMI, therefore, should be considered explorative.

In this study among 1781 postmenopausal women with HR+ BC, we have shown that obese patients experienced an increased risk of BCSM irrespective of age. These findings highlight the need for maintaining a healthy BMI in all patients with HR+ BC. In addition, we did not observe a reduced efficacy of extended anastrozole therapy in overweight and obese patients. Therefore, we conclude that (extended) aromatase inhibitor therapy can also be considered in overweight and obese patients with HR+ BC.

## Supplementary Material

pkad092_Supplementary_Data

## Data Availability

Study data underlying this article will be made easily available to any request to the corresponding author.
