# Supplementary material for: The prognostic and predictive effect of body mass index in hormone receptor-positive breast cancer
Source: JNCI Cancer Spectr. 2023 Nov 22;7(6):pkad092. doi: 10.1093/jncics/pkad092 (PMC10697786; doi:10.1093/jncics/pkad092)
Supplement: pkad092_Supplementary_Data [file pkad092_supplementary_data.pdf]

Supplementary materials

Supplement to: Lammers S.W.M., Geurts S.M.E., van Hellemond I.E.G. et al. The prognostic and predictive effect of body mass index in hormone receptor-positive breast cancer.

Corresponding author:
Vivianne C.G. Tjan-Heijnen, MD, PhD
ORCiD ID: 0000-0002-3935-7440
Department of Medical Oncology, Maastricht University Medical Centre
P.O. Box 5800, 6202 AZ Maastricht, the Netherlands
Email: vcg.tjan.heijnen@mumc.nl
Phone: +31433877025

Table of contents

Supplementary Table 1. Baseline characteristics of study participants aged <60 years at randomisation according to BMI class (N (%)) 3

Supplementary Table 2. Baseline characteristics of study participants aged ≥60 years at randomisation according to BMI class (N (%)) 4

Supplementary Table 3. Baseline characteristics of normal weight patients who were disease-free at 3 years after randomisation according to assigned treatment (N (%)) 5

Supplementary Table 4. Baseline characteristics of overweight patients who were disease-free at 3 years after randomisation according to assigned treatment (N (%)) 6

Supplementary Table 5. Baseline characteristics of obese patients who were disease-free at 3 years after randomisation according to assigned treatment (N (%)) 7

Supplementary Table 6. Multivariable analysis evaluating whether BMI is an independent prognostic factor for disease-free survival, accompanying Table 3 of the main manuscript 8

Supplementary Table 7. Multivariable analysis evaluating whether BMI is an independent prognostic factor for overall survival, accompanying Table 3 of the main manuscript 9

Supplementary Table 8. Multivariable analysis evaluating whether BMI is an independent prognostic factor for breast cancer-specific mortality, accompanying Table 3 of the main manuscript 10

Supplementary Table 9. Multivariable analysis evaluating whether BMI is an independent prognostic factor for other-cause mortality, accompanying Table 3 of the main manuscript 11

Supplementary Figure 1. Multivariable analyses of adapted overall survival evaluating the efficacy of 6 versus 3 years of anastrozole in the total study population and age subgroups (<60 years versus ≥60 years), stratified by BMI class at randomisation 12

Supplementary Table 1. Baseline characteristics of study participants aged <60 years at randomisation according to BMI class (N (%))

	Normal weight (N = 449)	Overweight (N = 392)	Obese (N = 182)	P for trend
Age at randomisation				0.002
Median – years (IQR)	51 (48 – 54)	52 (49 – 56)	53 (49 – 57)	
History of cardiovascular disease				<0.001
Yes	60 (13)	111 (28)	76 (42)	
Smoking history				0.42
Non-smoker	200 (46)	178 (47)	74 (41)	
Previous or current smoker	238 (54)	205 (54)	106 (59)	
Tumour classification				0.02
pT1	217 (48)	160 (41)	71 (39)	
pT2	186 (42)	198 (51)	87 (48)	
pT3/4	45 (10)	34 (9)	24 (13)	
Nodal status				0.58
Negative	136 (30)	111 (28)	52 (29)	
Positive	313 (70)	281 (72)	130 (71)	
Histological grade				0.71
Grade I	86 (20)	55 (14)	37 (21)	
Grade II	211 (48)	203 (53)	89 (51)	
Grade III	139 (32)	123 (32)	49 (28)	
Hormone receptor status				0.28
ER and PR positive	358 (80)	301 (77)	139 (76)	
ER or PR positive	91 (20)	91 (23)	43 (24)	
HER2 status				0.12
Positive	11 (3)	18 (5)	8 (5)	
Negative	409 (97)	348 (95)	158 (95)	
Histology				0.58
Lobular	88 (20)	56 (14)	36 (20)	
Other	361 (80)	336 (86)	146 (80)	
Breast-conserving surgery				0.39
Yes	219 (49)	200 (51)	95 (52)	
Prior (neo)adjuvant chemotherapy				0.43
Yes	414 (92)	369 (94)	170 (93)	
Previous duration of tamoxifen				0.62
Median – years (IQR)	2.3 (2.1 – 2.5)	2.3 (2.1 – 2.5)	2.3 (2.1 – 2.5)	
Recommended treatment duration of anastrozole				0.64
3 years	232 (52)	186 (47)	93 (51)	
6 years	217 (48)	206 (53)	89 (49)	

Abbreviations: BMI = body mass index; ER = oestrogen receptor; HER2 = human epidermal growth factor receptor 2; IQR = interquartile range; PR = progesterone receptor.

Percentages may exceed 100% because of rounding.

Missing values: smoking history (n = 22), tumour status (n = 1), histological grade (n = 31), and HER2 status (n = 71).

Supplementary Table 2. Baseline characteristics of study participants aged ≥60 years at randomisation according to BMI class (N (%))

	Normal weight (N = 229)	Overweight (N = 320)	Obese (N = 209)	P for trend
Age at randomisation				0.28
Median – years (IQR)	65 (62 – 71)	66 (62 – 72)	66 (62 – 71)	
History of cardiovascular disease				<0.001
Yes	87 (38)	142 (44)	128 (61)	
Smoking history				0.61
Non-smoker	111 (50)	164 (52)	108 (53)	
Previous or current smoker	110 (50)	152 (48)	97 (47)	
Tumour classification				0.16
pT1	113 (50)	146 (46)	88 (42)	
pT2	103 (45)	157 (49)	109 (52)	
pT3/4	12 (5)	17 (5)	12 (6)	
Nodal status				0.88
Negative	82 (36)	126 (39)	73 (35)	
Positive	147 (64)	194 (61)	136 (65)	
Histological grade				0.64
Grade I	36 (16)	58 (18)	31 (16)	
Grade II	115 (52)	164 (52)	108 (55)	
Grade III	72 (32)	94 (30)	57 (29)	
Hormone receptor status				0.14
ER and PR positive	159 (69)	220 (69)	159 (76)	
ER or PR positive	70 (31)	100 (31)	50 (24)	
HER2 status				0.17
Positive	4 (2)	2 (1)	1 (1)	
Negative	209 (98)	287 (99)	191 (100)	
Histology				0.34
Lobular	47 (21)	48 (15)	51 (24)	
Other	182 (80)	272 (85)	158 (76)	
Breast-conserving surgery				0.97
Yes	112 (49)	151 (47)	102 (49)	
Prior (neo)adjuvant chemotherapy				0.28
Yes	71 (31)	109 (34)	75 (36)	
Previous duration of tamoxifen				0.65
Median – years (IQR)	2.3 (2.1 – 2.5)	2.2 (2.1 – 2.5)	2.3 (2.1 – 2.5)	
Recommended treatment duration of anastrozole				0.22
3 years	105 (46)	158 (49)	108 (52)	
6 years	124 (54)	162 (51)	101 (48)	

Abbreviations: BMI = body mass index; ER = oestrogen receptor; HER2 = human epidermal growth factor receptor 2; IQR = interquartile range; PR = progesterone receptor.

Percentages may exceed 100% because of rounding.

Missing values: smoking history (n = 16), tumour status (n = 1), histological grade (n = 23), and HER2 status (n = 64).

Supplementary Table 3. Baseline characteristics of normal weight patients who were disease-free at 3 years after randomisation according to assigned treatment (N (%))

		6-year anastrozole (N = 306)	3-year anastrozole (N = 307)	P-value
Age at randomisation				0.13
	Median – years (IQR)	55 (51 – 62)	54 (50 – 61)	
Tumour status				0.49
	pT1	156 (51)	142 (46)	
	pT2	126 (41)	137 (45)	
	pT3/4	23 (8)	27 (9)	
Nodal status				0.37
	Node-negative	102 (33)	113 (37)	
	Node-positive	204 (67)	194 (63)	
Histological grade				0.19
	Grade I	50 (17)	68 (23)	
	Grade II	155 (52)	142 (48)	
	Grade III	92 (31)	89 (30)	
Hormone receptor status				0.69
	ER and PR positive	233 (76)	238 (78)	
	ER or PR positive	73 (24)	69 (23)	
HER2 status				0.26
	Positive	5 (2)	9 (3)	
	Negative	284 (98)	272 (97)	
Histology				0.70
	Lobular	60 (20)	64 (21)	
	Other	246 (80)	243 (79)	
Breast-conserving surgery				0.44
	Yes	158 (52)	149 (49)	
Prior (neo)adjuvant chemotherapy				0.23
	Yes	217 (71)	231 (75)	
Previous duration of tamoxifen				0.35
	Median – years (IQR)	2.2 (2.1 – 2.5)	2.3 (2.1 – 2.6)	
Abbreviations: ER = oestrogen receptor; HER2 = human epidermal growth factor receptor 2; IQR = interquartile range; PR = progesterone receptor. Percentages may exceed 100% because of rounding.				
Missing values: tumour status (n = 2), histological grade (n = 17) and HER2 status (n = 43).				

Supplementary Table 4. Baseline characteristics of overweight patients who were disease-free at 3 years after randomisation according to assigned treatment (N (%))

		6-year anastrozole (N = 324)	3-year anastrozole (N = 304)	P-value
Age at randomisation				0.93
	Median – years (IQR)	58 (52 – 64)	58 (52 – 65)	
Tumour status				0.64
	pT1	139 (43)	138 (45)	
	pT2	164 (51)	143 (47)	
	pT3/4	21 (7)	23 (8)	
Nodal status				0.25
	Node-negative	123 (38)	102 (34)	
	Node-positive	201 (62)	202 (66)	
Histological grade				0.85
	Grade I	52 (16)	53 (18)	
	Grade II	167 (53)	157 (53)	
	Grade III	98 (31)	87 (29)	
Hormone receptor status				0.57
	ER and PR positive	243 (75)	222 (73)	
	ER or PR positive	81 (25)	82 (27)	
HER2 status				0.93
	Positive	9 (3)	9 (3)	
	Negative	285 (97)	273 (97)	
Histology				0.40
	Lobular	48 (15)	38 (13)	
	Other	276 (85)	266 (88)	
Breast-conserving surgery				0.21
	Yes	173 (53)	147 (48)	
Prior (neo)adjuvant chemotherapy				0.41
	Yes	215 (66)	211 (69)	
Previous duration of tamoxifen				0.53
	Median – years (IQR)	2.2 (2.1 – 2.5)	2.3 (2.1 – 2.5)	

Abbreviations: ER = oestrogen receptor; HER2 = human epidermal growth factor receptor 2; IQR = interquartile range; PR = progesterone receptor.

Percentages may exceed 100% because of rounding.

Missing values: histological grade (n = 14) and HER2 status (n = 52).

Supplementary Table 5. Baseline characteristics of obese patients who were disease-free at 3 years after randomisation according to assigned treatment (N (%))

		6-year anastrozole (N = 167)	3-year anastrozole (N = 181)	P-value
Age at randomisation				0.91
	Median – years (IQR)	60 (53 – 66)	61 (52 – 67)	
Tumour status				0.35
	pT1	65 (39)	84 (46)	
	pT2	88 (53)	82 (45)	
	pT3/4	14 (8)	15 (8)	
Nodal status				0.04
	Node-negative	47 (28)	70 (39)	
	Node-positive	120 (72)	111 (61)	
Histological grade				0.14
	Grade I	33 (21)	34 (19)	
	Grade II	89 (57)	87 (50)	
	Grade III	33 (21)	54 (31)	
Hormone receptor status				0.88
	ER and PR positive	128 (77)	140 (77)	
	ER or PR positive	39 (23)	41 (23)	
HER2 status				0.48
	Positive	2 (1)	4 (2)	
	Negative	149 (99)	163 (98)	
Histology				0.07
	Lobular	42 (25)	31 (17)	
	Other	125 (75)	150 (83)	
Breast-conserving surgery				0.83
	Yes	85 (51)	90 (50)	
Prior (neo)adjuvant chemotherapy				0.20
	Yes	109 (65)	106 (59)	
Previous duration of tamoxifen				0.57
	Median – years (IQR)	2.3 (2.1 – 2.5)	2.2 (2.1 – 2.5)	

Abbreviations: ER = oestrogen receptor; HER2 = human epidermal growth factor receptor 2; IQR = interquartile range; PR = progesterone receptor.

Percentages may exceed 100% because of rounding.

Missing values: histological grade (n = 18) and HER2 status (n = 30).

Supplementary Table 6. Multivariable analysis evaluating whether BMI is an independent prognostic factor for disease-free survival, accompanying Table 3 of the main manuscript

Variables	HR	95% CI	P-value
BMI			
Overweight vs normal weight	1.16	0.97 – 1.38	0.10
Obese vs normal weight	1.26	1.03 – 1.54	0.03
Age			
≥60 years vs <60 years	1.63	1.34 – 1.98	<0.001
History of cardiovascular disease			
Yes vs no	1.18	1.01 – 1.39	0.04
Smoking history			
Yes vs no	1.26	1.09 – 1.46	0.002
Tumour status			
pT2 and pT3/4 vs pT1	1.37	1.17 – 1.60	<0.001
Nodal status			
pN positive vs pN negative	1.56	1.31 – 1.85	<0.001
Histology			
Lobular vs other	1.26	1.04 – 1.51	0.02
Histological grade			
Grade 3 vs grade 1 and 2	1.46	1.24 – 1.72	<0.001
Hormone receptor status			
Single HR+ vs double HR+	1.05	0.89 – 1.24	0.58
Previous chemotherapy			
Yes vs no	0.81	0.66 – 0.99	0.04

Abbreviations: BMI = body mass index; CI = confidence interval; HR = hazard ratio; HR+ hormone receptor-positive.

Supplementary Table 7. Multivariable analysis evaluating whether BMI is an independent prognostic factor for overall survival, accompanying Table 3 of the main manuscript

**Variables**	**HR**	**95% CI**	**P-value**
BMI			
Overweight vs normal weight	1.20	0.97 – 1.48	0.10
Obese vs normal weight	1.16	0.91 – 1.48	0.23
Age			
≥60 years vs <60 years	1.97	1.55 – 2.49	<0.001
History of cardiovascular disease			
Yes vs no	1.27	1.05 – 1.53	0.02
Smoking history			
Yes vs no	1.20	1.00 – 1.43	0.05
Tumour status			
pT2 and pT3/4 vs pT1	1.48	1.22 – 1.78	<0.001
Nodal status			
pN positive vs pN negative	1.71	1.39 – 2.10	<0.001
Histology			
Lobular vs other	1.36	1.09 – 1.70	0.007
Histological grade			
Grade 3 vs grade 1 and 2	1.67	1.37 – 2.04	<0.001
Hormone receptor status			
Single HR+ vs double HR+	1.09	0.89 – 1.33	0.42
Previous chemotherapy			
Yes vs no	0.73	0.58 – 0.93	0.01

Abbreviations: BMI = body mass index; CI = confidence interval; HR = hazard ratio; HR+ hormone receptor-positive.

Supplementary Table 8. Multivariable analysis evaluating whether BMI is an independent prognostic factor for breast cancer-specific mortality, accompanying Table 3 of the main manuscript

Variables	sHR	95% CI	P-value
BMI			
Overweight vs normal weight	1.25	0.93 – 1.68	0.15
Obese vs normal weight	1.36	0.97 – 1.91	0.07
Age			
≥60 years vs <60 years	1.80	1.30 – 2.48	<0.001
History of cardiovascular disease			
Yes vs no	0.91	0.68 – 1.21	0.50
Smoking history			
Yes vs no	1.05	0.82 – 1.36	0.69
Tumour status			
pT2 and pT3/4 vs pT1	1.52	1.16 – 1.99	0.003
Nodal status			
pN positive vs pN negative	2.24	1.64 – 3.06	<0.001
Histology			
Lobular vs other	1.44	1.06 – 1.95	0.02
Histological grade			
Grade 3 vs grade 1 and 2	2.05	1.56 – 2.70	<0.001
Hormone receptor status			
Single HR+ vs double HR+	1.08	0.81 – 1.45	0.60
Previous chemotherapy			
Yes vs no	1.18	0.84 – 1.66	0.34

Abbreviations: BMI = body mass index; CI = confidence interval; HR+ hormone receptor-positive; sHR = subdistribution hazard ratio.

Supplementary Table 9. Multivariable analysis evaluating whether BMI is an independent prognostic factor for other-cause mortality, accompanying Table 3 of the main manuscript

Variables	sHR	95% CI	P-value
BMI			
Overweight vs normal weight	1.12	0.83 – 1.52	0.45
Obese vs normal weight	1.01	0.71 – 1.43	0.96
Age			
≥60 years vs <60 years	1.99	1.38 – 2.88	<0.001
History of cardiovascular disease			
Yes vs no	1.72	1.32 – 2.25	<0.001
Smoking history			
Yes vs no	1.35	1.04 – 1.74	0.02
Tumour status			
pT2 and pT3/4 vs pT1	1.25	0.96 – 1.62	0.10
Nodal status			
pN positive vs pN negative	1.17	0.88 – 1.56	0.28
Histology			
Lobular vs other	1.18	0.85 – 1.63	0.32
Histological grade			
Grade 3 vs grade 1 and 2	1.14	0.85 – 1.51	0.39
Hormone receptor status			
Single HR+ vs double HR+	1.07	0.80 – 1.43	0.65
Previous chemotherapy			
Yes vs no	0.50	0.35 – 0.71	<0.001

Abbreviations: BMI = body mass index; CI = confidence interval; HR+ hormone receptor-positive; sHR = subdistribution hazard ratio.

Supplementary Figure 1. Multivariable analyses of adapted overall survival evaluating the efficacy of 6 versus 3 years of anastrozole in the total study population and age subgroups (<60 years versus ≥60 years), stratified by BMI class at randomisation

	Number of patients		Number of events		HR[a] (95% CI)	P-value	10-year adapted overall survival (95% CI)		P interaction
	6-year	3-year	6-year	3-year			6-year	3-year	
**All patients (n = 1589)**									
BMI group (kg/m^2)									0.03
18.5-24.9 (normal weight)	306	307	57	38	1.48 (0.98-2.23)	0.06	82.2 (77.2-86.2)	87.5 (83.1-90.8)	
25.0-29.9 (overweight)	324	304	23	27	0.72 (0.51-1.02)	0.07	81.4 (76.6-85.3)	76.2 (70.8-80.7)	
≥30 (obese)	167	181	44	49	0.92 (0.60-1.41)	0.72	74.2 (66.4-80.4)	73.1 (65.6-79.2)	
**<60 years (n = 932)**									
BMI group (kg/m^2)									0.62
18.5-24.9 (normal weight)	203	214	22	17	1.26 (0.66-2.39)	0.48	89.8 (84.4-93.5)	91.9 (87.2-94.9)	
25.0-29.9 (overweight)	182	170	23	22	0.96 (0.53-1.74)	0.89	87.7 (81.9-91.7)	87.2 (81.0-91.5)	
≥30 (obese)	79	84	14	16	0.74 (0.35-1.55)	0.42	83.2 (72.8-89.9)	82.9 (72.9-89.5)	
**≥60 years (n = 657)**									
BMI group (kg/m^2)									0.02
18.5-24.9 (normal weight)	103	93	35	21	1.61 (0.93-2.80)	0.09	67.2 (57.0-75.5)	77.2 (66.7-84.8)	
25.0-29.9 (overweight)	142	134	38	50	0.62 (0.40-0.95)	0.03	73.0 (64.5-79.8)	62.0 (52.8-70.0)	
≥30 (obese)	88	97	30	33	0.87 (0.51-1.48)	0.60	65.2 (52.8-75.0)	64.4 (53.2-73.6)	

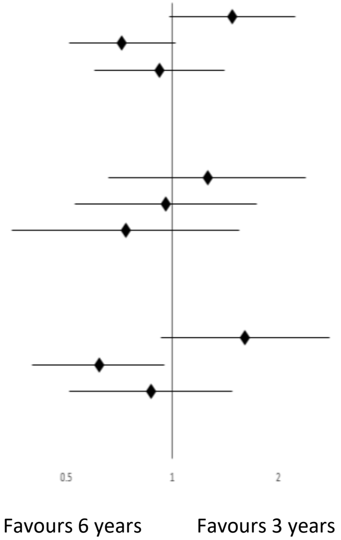

Abbreviations: BMI = body mass index; CI = confidence interval; HR = hazard ratio.

[a]Analyses were adjusted for age (≥60 years versus <60 years), history of cardiovascular disease (yes versus no), smoking history (yes versus no), tumour status (≥pT2 versus pT1), nodal status (pN positive versus pN negative), histology (lobular versus other), histological grade (histological grade 3 versus histological grade 1 and 2), hormone receptor status (oestrogen receptor-positive or progesterone receptor-positive versus oestrogen receptor-positive and progesterone receptor-positive), and previous chemotherapy (yes versus no). Age was excluded as a confounding factor in the stratified analyses by age.
